# A study of National Health Service management of chronic osteoarthritis and low back pain

**DOI:** 10.1017/S1463423614000140

**Published:** 2014-03-27

**Authors:** Oliver R. Hart, Ruth M. Uden, James E. McMullan, Mark S. Ritchie, Timothy D. Williams, Blair H. Smith

**Affiliations:** 1 General Practitioner, Sloan Medical Practice, Sheffield, England; 2 Management Consultant, pH Associates, Marlow, England; 3 General Practitioner, Tynan Surgery, Tynan, Armagh, Northern Ireland; 4 General Practitioner, Sway Road Surgery, Morriston, Wales; 5 General Practitioner, Sothall Medical Practice, Sheffield, England; 6 Professor, Department of Population Science, University of Dundee, Dundee, Scotland; 7 General Practitioner, Peterhead Health Centre, Peterhead, Scotland

**Keywords:** analgesic prescribing, low back pain, osteoarthritis, pain, primary healthcare, referral and consultations

## Abstract

**Aim:**

To describe treatment and referral patterns and National Health Service resource use in patients with chronic pain associated with low back pain or osteoarthritis, from a Primary Care perspective.

**Background:**

Osteoarthritis and low back pain are the two commonest debilitating causes of chronic pain, with high health and social costs, and particularly important in primary care. Understanding current practice and resource use in their management will inform health service and educational requirements and the design and optimisation of future care.

**Method:**

Multi-centre, retrospective, descriptive study of adults (⩾18 years) with chronic pain arising from low back pain or osteoarthritis, identified through primary care records. Five general practices in Scotland, England (two), Northern Ireland and Wales. All patients with a diagnosis of low back pain or osteoarthritis made on or before 01/09/2006 who had received three or more prescriptions for pain medication were identified and a sub-sample randomly selected then consented to an in-depth review of their medical records (*n*=264). Data on management of chronic pain were collected retrospectively from patients’ records for three years from diagnosis (‘newly diagnosed’ patients) or for the most recent three years (‘established’ patients).

**Findings:**

Patients received a wide variety of pain medications with no overall common prescribing pattern. GP visits represented the majority of the resource use and ‘newly diagnosed’ patients were significantly more likely to visit their GP for pain management than ‘established’ patients. Although ‘newly diagnosed’ patients had more referrals outside the GP practice, the number of visits to secondary care for pain management was similar for both groups.

**Conclusion:**

This retrospective study confirmed the complexity of managing these causes of chronic pain and the associated high resource use. It provides an in-depth picture of prescribing and referral patterns and of resource use.

## Introduction

The International Association for the Study of Pain defines chronic pain as ‘pain that has persisted beyond normal tissue healing time’, and in the absence of rigorous markers for normal tissue healing time, a period of three months is usually accepted as the point at which pain can be classified as chronic (Merskey and Bogduk, 1994). Approximately 20% of the adult population in Europe have been found to be suffering from chronic pain (Breivik *et al.*, 2006), with over 5% experiencing severe, disabling pain (Smith *et al.*, 2001). The management of chronic pain represents a significant burden to the National Health Service (NHS); it has been estimated that chronic pain accounts for 4.6 million general practice appointments in the United Kingdom each year, at a cost of £69 million, equivalent to 793 full-time general practitioners (GPs) (Belsey, 2002). Osteoarthritis (OA) and low back pain (LBP) contribute significantly to the number of people in the United Kingdom with chronic pain, together accounting for more than half of all cases (Elliott *et al.*, 1999) and with the ageing population, the burden is likely to increase over the coming years.

There is little rigorous evidence to guide the day-to-management of chronic pain in primary care (Smith *et al.*, 2010), and outside the clinical trial setting, current primary care treatment patterns and referral rates in chronic pain management are not well understood. It is therefore difficult to target resources and identify educational needs for this major primary care disease burden, and to quantify ways in which pain management may be improved. This study aimed to describe the management of patients with OA and chronic LBP in real-world primary care practice and to quantify the NHS resource utilisation associated with the management of these conditions.

## Methods

### Design

A multi-centre, retrospective, descriptive observational study.

### Setting

Five general practices were purposively selected [Scotland, England (two), Wales and Northern Ireland], to provide a range of practice size (5200–18 000 patients/practice), number of GPs per practice (3–11 GPs/practice; total 34 GPs) and a mixture of urban and rural locations and socioeconomic groups of patients. One GP in each practice was identified as a Principal Investigator (PI) on the basis of his interest in chronic pain.

### Patient identification

Using the practice database, the PI at each site identified all patients with a diagnosis of LBP or OA (based on Read coding) on or before 01/09/2006, aged ⩾18 years, without a diagnosis of cancer-related pain, and who had received three or more prescriptions for pain medication. Of these, 250 patients at each practice were randomly selected and invited to consent to the study and have a researcher review their medical records. We identified individuals with clinically significant chronic pain (Smith *et al.*, 2001), as those who had received at least three prescriptions for any pain medication since diagnosis, based on a previously validated search protocol (McDermott *et al.*, 2006). Consenting patients who met the inclusion criteria of having a diagnosis of OA or LBP and who had received at least three prescriptions for any pain medication since diagnosis, were therefore included in the study (*n*=264).

### Data collection and analysis

Data were collected from two cohorts of patients: in ‘newly diagnosed’ patients (ie OA or LBP diagnosed between 01/09/2004 and 01/09/2006) data from the first three years after diagnosis were collected to describe the initial stages of management; in ‘established disease’ patients (ie diagnosed before 01/09/2004) data from the most recent three years were collected to provide data on recent pain management, later in the course of the condition.

Anonymised data relating to analgesic prescriptions, consultations, and referrals between 01/09/2004 and 01/09/2009 were collected from primary care paper records and electronic systems between April and July 2010 by researchers working to data collection guidelines.

Data were collected on pain medications prescribed at least once by GPs or Nurse Prescribers. The drugs included analgesics, antidepressants and anti-epileptics where the clinical record clearly showed that the prescription was for pain.

Opioid analgesics were classified as ‘strong’ or ‘weak’ according to the *British National Formulary* (British Medical Association and the Royal Pharmaceutical Society of Great Britain, 2010). Weak opioids identified during the study included codeine, dihydrocodeine, meptazinol and tramadol, and ‘strong’ included buprenorphine, fentanyl, morphine and oxycodone.

All visits to the GP practice were recorded during the study period. Visits were classified as ‘pain related’ or ‘non-pain related’ according to the presence or absence of reference to pain in the clinical record.

All records of patient referrals that occurred within the study period, from the GP to secondary care or non-NHS services for pain management were noted.

Non-drug interventions were defined as interventions related to the management of pain but did not involve the administration of medication, and were recorded in the notes as recommended or administered to the patient.

Co-prescribed medication were defined as medication to prevent or manage unwanted effects of pain medication (eg laxatives, anti-emetics, medication for indigestion).

Analysis was undertaken in MS Excel and SPSS for Windows. Statistical analysis employed the Fisher Exact Test of Probability and the Kruskal–Wallis test.

Ethical approval was obtained from Outer South East London Research Ethics Committee (09/HO805/42) and NHS R&D approval was obtained in each study area.

## Results

### Study sample

From a total of 1250 identified patients, 606 (49%) consented and 264 (44%) of these were eligible. Reasons for ineligibility were: at least three prescriptions not issued (*n*=140), and incorrect or late completion of the consent form (*n*=202).

The sample was 71% female and the mean current age was 62 years in ‘newly diagnosed’, and 66 years in ‘established’ patients (Table 1). Almost two-thirds (64%) of patients had OA, although among ‘newly diagnosed’ patients the split between OA and LBP was more even (55% OA). OA or LBP was diagnosed a mean of 6.7 years before data collection (one year for ‘newly diagnosed’ and 8.1 years for ‘established’ patients). No patients in the study sample had a recorded diagnosis of both OA and LBP. The earliest date of diagnosis was 1977.

**Table 1:** Description of study sample and drug treatments for pain

	Newly diagnosed (*n*=53) [*N* (%)]	Established (*n*=211) [*N* (%)]	All (*n*=264) [*N* (%)]
Mean current age (years)	62	66	65
Male^a^	15 (28)	60 (29)	75 (29)
Female	38 (72)	150 (71)	188 (71)
Low back pain	24 (45)	72 (34)	96 (36)
Osteoarthritis pain	29 (55)	139 (66)	168 (64)
Mean time since diagnosis of chronic pain (years)	1.0	8.1	6.7
			

a Sex of one patient not recorded.

### Pain treatment

#### Prescribed medication

**Figure 1:**
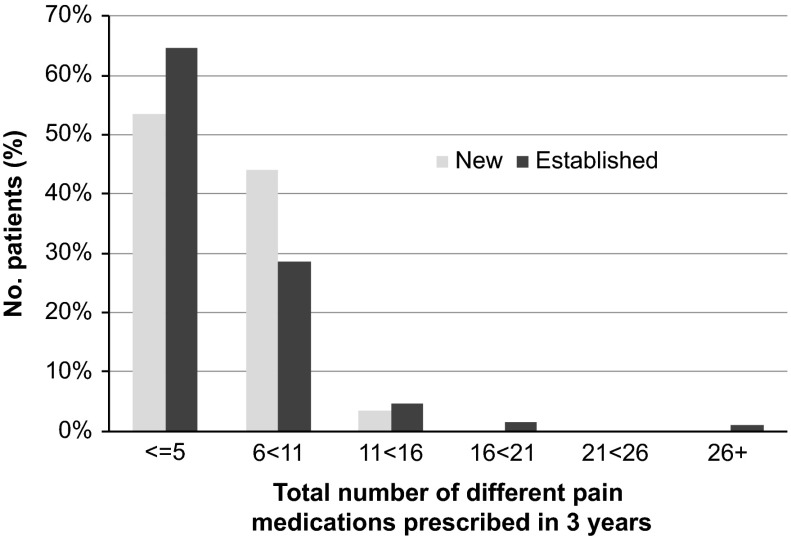
Number of pain medications prescribed in three years

Most patients (62%) were prescribed five or fewer different drugs at least once for pain management over the three-year study period and a significant minority (38%) received six or more different drugs (Figure 1). Similarly, while many patients (45%) had received no co-prescribed drugs, most (49%) received between one and three drugs prescribed for unwanted effects of pain medication. Differences in these proportions between ‘newly diagnosed’ and ‘established’ were not statistically significant.

‘Weak’ opioids were prescribed to 161 (61%) patients and the majority of these patients received a tramadol product (122, 46%; Table 2). Compound analgesics containing ‘weak’ opioids and paracetamol were prescribed to 194 (73%) patients. Almost all patients (96%) received an opioid containing analgesic within the three-year study period and 17% of patients (*n*=44) were prescribed at least one type of ‘strong opioid’: [*n*=7 (13%) ‘newly diagnosed’ and *n*=37 (18%) ‘established’ patients]: 26 (10%) received buprenorphine, 14 (5%) morphine, 10 (4%) fentanyl patch and 9 (4%) oxycodone (Table 3).

**Table 2:** Drug treatments for pain

	Newly diagnosed (*n*=53) [*N* (%)]	Established (*n*=211) [*N* (%)]	All (*n*=264) [*N* (%)]
Non-opioid	48 (91)	181 (86)	229 (87)
Paracetamol	30 (57)	100 (47)	130 (49)
Systemic NSAID	35 (66)	118 (56)	153 (58)
Topical NSAID	16 (30)	68 (32)	84 (32)
COX-II inhibitor	6 (11)	15 (7)	21 (8)
Other non-opioid analgesic	4 (8)	8 (4)	12 (5)
Opioid	53 (100)	201 (95)	254 (96)
Compound analgesic (containing weak opioid)	41 (77)	153 (73)	194 (73)
Weak opioid analgesic	35 (66)	126 (60)	161 (61)
Strong opioid analgesic	7 (13)	37 (18)	44 (17)
Adjuvant analgesic drugs (eg amitriptyline and pregabalin)	33 (62)	121 (57)	154 (58)

NSAID=non-steroidal anti-inflammatory drugs; COX-II=cyclooxygenase-II. All drugs grouped by *British National Formulary* (British Medical Association and the Royal Pharmaceutical Society of Great Britain, 2010) classification.

**Table 3:** Opioid and co-prescribed medications

	Newly diagnosed (*n*=53) [*N* (%)]	Established (*n*=211) [*N* (%)]	All (*n*=264) [*N* (%)]
Strong opioid analgesic	7 (13)	37 (18)	44 (17)
Buprenorphine^a^	3 (6)	23 (11)	26 (10)
Morphine	3 (6)	11 (5)	14 (5)
Fentanyl patch	1 (2)	9 (4)	10 (4)
Oxycodone	0	9 (4)	9 (4)
Weak opioid analgesic	35 (66)	126 (60)	161 (61)
Tramadol^b^	26 (49)	84 (40)	110 (42)
Tramadol and paracetamol	–	12 (6)	12 (4)
Codeine	9 (17)	27 (13)	36 (14)
Dihydrocodeine	10 (19)	24 (11)	34 (13)
Meptazinol	0	1 (0.5)	1 (0.4)
Co-prescribed medication	31 (58)	114 (54)	145 (55)
Laxative	27 (51)	81 (38)	108 (41)
Gastro-protective agent	27 (51)	101 (48)	128 (48)
Anti-emetic	4 (8)	26 (12)	30 (11)

Opioids have been classified according to *British National Formulary* (BNF) (British Medical Association and the Royal Pharmaceutical Society of Great Britain, 2010).

a Buprenorphine is classified as a strong opioid analgesic, but it is recognised that low dose patches may be included and would more appropriately be classified as weak opioids. As dose was not recorded in this study drugs cannot be presented by strength.

b Tramadol and Tramacet are classified as weak opioids in BNF 59; it is recognised that tramadol is only considered a strong opioid at high doses (⩾400 mg daily), also that the maximum recommended daily dose of Tramacet includes 300 mg/day of tramadol.

Oral non-steroidal anti-inflammatory drugs (NSAIDs) were prescribed to 58% of patients [35 (66%) ‘newly diagnosed’ and 118 (56%) ‘established’ patients]. Adjuvant analgesic drugs such as tricyclic antidepressants and anti-epileptics were prescribed for 154 patients (58%). The most prevalent adjuvant analgesic prescribed was amitriptyline, to 80 (30%) patients, followed by pregabalin and gabapentin to 29 (11%) and 26 (10%) of patients, respectively.

Co-prescribed medication to prevent or manage unwanted effects of pain medication was prescribed to 145 (55%) patients, including laxatives (41%), gastro-protective agents (48%) and anti-emetics (11%) (Table 3). Rates of co-prescribing were similar among ‘newly diagnosed’ and ‘established’ patients.

Reasons for changing pain medication were documented in only 457 (21%) of the 2188 recorded changes over the study period.

#### Non-drug interventions

Non-drug interventions such as physiotherapy and acupuncture were prescribed for 34 (64%) ‘newly diagnosed’ and 86 (41%) ‘established’ patients during the three-year study period. Overall, 105 (40%) patients were recorded as receiving physiotherapy during the study period [29 (55%) ‘newly diagnosed’ and 76 (35%) ‘established’]. Exercises were recommended and recorded by the GP for 6% overall (17% ‘newly diagnosed’, 4% ‘established’). Acupuncture was similarly recommended and recorded for 6% overall (8% ‘newly diagnosed’, 6% ‘established’) and transcutaneous electrical nerve stimulation for 3% overall (4% ‘newly diagnosed’, 2% ‘established’). One episode each of psychotherapy, occupational therapy, osteopathy and heat pads was recorded.

#### Referral patterns

Table 4 shows the number of patients referred outside the GP practice for investigation, treatment or specialist opinion during the study period, and the specialties to which they were referred. Eighty-one per cent of ‘newly diagnosed’ patients and 68% of ‘established’ patients were referred elsewhere for pain management, with a significantly higher referral rate for ‘newly diagnosed’ patients [0.77 versus 0.62 referrals/patient/year (*P*<0.01)]. However, ‘established’ patients were referred to a wider range of specialties and providers. In ‘newly diagnosed’ patients where data were available, the mean (SD) time from diagnosis to first referral was 9.4 (10.0) months (range 0–34.3). Physiotherapy was the most common referral (55% ‘newly diagnosed’ patients) with a mean of 0.26 referrals/patient/year over the first three years since diagnosis.

**Table 4:** Referrals for pain management

Referral	Newly diagnosed (*n*=53) [*N* (%)]	Established (*n*=211) [*N* (%)]	All (*n*=264) [*N* (%)]
No referral	10 (19)	67 (32)	77 (29)
Referral	43 (81)	144 (68)	187 (71)
Therapy and investigation			
Physiotherapy	29 (55)	74 (35)	103 (40)
Radiology	22 (42)	77 (37)	99 (37)
Secondary care specialist			
Orthopaedics	20 (38)	60 (28)	80 (30)
Pain clinic	2 (4)	19 (9)	21 (8)
Rheumatology	7 (13)	9 (4)	16 (6)
Neurosurgery	1 (2)	8 (4)	9 (3)
Neurology	0	6 (3)	6 (2)
Other referral sites^a^	16 (30)	95 (45)	111 (42)
Referral rate	0.77 referrals/patient/year	0.62 referrals/patient/year	

a Other referral sites included: musculoskeletal clinic, podiatry, geriatrics, intermediate care, acupuncture, anaesthetics, counselling, day procedure unit, falls prevention, foot and ankle service, Nurse, occupational therapy, pathology, urology, mental health.

#### Resource use

NHS resources in primary and secondary care were heavily used by these patients (Table 5).

**Table 5:** Distribution of visits to National Health Service services per patient per year for ‘newly diagnosed’ and ‘established’ patients

	‘Newly diagnosed’ (*n*=53)						‘Established’ (*n*=211)		
	Visits per patient in first year after diagnosis			Visits per patient year (3 years’ data annualised)			Visits per patient year (3 years’ data annualised)		
	No. patients attending [N (%)]	Median no. visits (range)	Mean no. visits (range)	No. patients attending [N (%)]	Median no. visits (range)	Mean no. visits (range)	No. patients attending [N (%)]	Median no. of visits (range)	Mean no. visits (range)
Pain-related GP visits	49 (92)	4.0 (0−12)	5.1 (0−12)	51 (96)	3.7 (0−11)	3.9 (0−11)	197 (93)	2.3 (0−21)	3.0 (0−21)
Non-pain-related GP visits	50 (94)	5.0 (0−37)	7.2 (0−37)	53 (100)	6.3 (0−27)	7.7 (0−27)	211 (100)	8.3 (0−43)	9.4 (0−43)
Physiotherapy	18 (34)	0.0 (0−11)	0.6 (0−11)	29 (55)	0.3 (0−5)	0.6 (0−5)	76 (36)	0.0 (0−7)	0.3 (0−7)
Outpatients	16 (30)	0.0 (0−4)	0.6 (0−4)	32 (60)	0.3 (0−4)	0.7 (0−4)	133 (63)	0.3 (0−7)	1.0 (0−7)
Inpatients	4 (8)	0.0 (0−1)	0.1 (0−1)	5 (9)	0.0 (0−0.3)	0.03 (0−0.3)	33 (16)	0.0 (0−2)	0.1 (0−2)
A&E	2 (4)	0.0 (0−1)	0.04 (0−1)	7 (13)	0.0 (0−0.3)	0.04 (0−0.3)	22 (10)	0.0 (0−1)	0.1 (0−1)
Day case	0	0	0	1 (2)	0.0 (0−0.3)	0.01 (0−0.3)	17 (8)	0.0 (0−3)	0.1 (0−3)

Most GP practice visits for pain management (67%) were with a GP, 29% were with a Practice Nurse and 4% were with other practice staff.

‘Newly diagnosed’ patients visited their GP practice significantly more frequently for pain-related reasons than did ‘established’ patients, both in the first year after diagnosis (median 4 visits) and in the average per year (3.7) among the ‘newly diagnosed’ patients, compared with 2.3 per year among ‘established’ patients (*P*<0.001). (Mean pain-related GP visits per year: 3.9 for ‘newly diagnosed’ and 3.0 for ‘established’.)

For non-pain-related visits, ‘established’ patients visited their practice significantly more frequently than ‘newly diagnosed’ patient. This was true in both the first year (median 5.0 visits) and the average per year (6.3), compared with 8.3 per year among ‘established’ patients (*P*<0.005). (Mean non-pain-related GP visits per year were 7.7 for ‘newly diagnosed’ and 9.4 for ‘established’ patients.)

Secondary care visits were less frequent than primary care visits. Outpatient visits to specialists were the most common, with no differences in frequency found between ‘newly diagnosed’ and ‘established’ patients.

## Discussion

This retrospective multi-centre study was designed to gather data on real-world clinical practice across the United Kingdom, and in particular to describe current treatment, referral patterns and NHS resource use associated with the management of chronic pain in OA and LBP. This is the first systematic recording of the management of chronic pain in routine general practice and provides a picture of the impact of chronic pain on primary care practice. The findings highlight both the large resource requirement among this group in terms of prescribing, consultation and referral, and the wide range of practice.

Of course, the results are not representative of all GP practices in the United Kingdom as patient populations and pain management vary greatly between practices and only five practices participated in the study. Each practice had a GP who was relatively well informed in pain-related issues, which may have influenced their approach to treating pain. These GPs may have a more proactive and confident approach to prescribing and referral of patients than GPs without this interest. Nevertheless, the study sample was selected from all the patients within these practices, managed by all 34 GPs, not just those managed by the GP with the interest in pain, and their overall influence on the results is likely to be very small.

In its retrospective design, the study data quality relied on the completeness of primary care clinical records. In addition, information on non-NHS treatments, for example, over-the-counter medications, complementary therapies and other private appointments were not available. It was not possible to identify all patients with chronic pain from clinical records, as there are no primary care registers or Read codes for patients with chronic pain. It is therefore unclear what proportion of OA and LBP patients with chronic pain were excluded from this study, and how their prescribing and referral patterns differed from those we included. It has previously been shown that seeking of treatment and use of analgesics identifies those with the most significant chronic pain (Smith *et al.*, 2001), and it is likely that we have included those of most importance to the health services. Breivik *et al.* (2006) found that 78% of all individuals reporting chronic pain had received a prescription of analgesic medication. We only included those with LBP and OA for logistical reasons, and while our findings cannot be directly extrapolated to those with other conditions, it is likely that they will be similar for other musculoskeletal causes of chronic pain.

The number of different pain medications given to patients in this study suggests that management of chronic pain associated with OA or LBP is complex in many patients, often involving a trial of several different analgesics and combinations with adjuvant pain medication, non-drug therapies, and in many cases, referral for specialist opinion, resulting in individualised patient management. The large proportion of patients receiving co-prescribed medication suggests that avoiding unwanted effects of pain medication is also important in the management of these patients. There is no evidence of a standard approach to chronic pain management in primary care. This highly individualised care seen in this study is in line with guidance from The National Institute for Health and Clinical Excellence (NICE, 2008; 2009; 2010) but does involve close patient management that the resource use data from this study also shows.

Patients with multi-morbidities also often require close management to avoid adverse effects from polypharmacy. NSAIDs and opioids are the ‘mainstay’ of chronic pain management and are included in the top 10 medication most associated with adverse-drug-reaction related hospital admissions (Pirmohamed *et al*., 2004). Describing the presence of co-morbidities was beyond the scope of this study, but a high prevalence of multi-morbidities in adult and older adult patients in primary care has recently been described; 87% of patients with painful conditions including back pain and OA had at least one co-morbid condition, 46% had three or more (Barnett *et al.*, 2012). In view of the high prevalence of both chronic pain and multi-morbidity, the complexity we have described in the management of chronic pain alone, and the poor tolerability of many pain medications, it would seem clear that robust guidelines for individualised care should be developed for primary care, to minimise morbidity and maximise patients’ quality of life. Ideally, these would address chronic pain in the context of its co-morbidities (Guthrie *et al.*, 2012).

The current relevant guidance emphasises the importance of identifying early the needs of patients with chronic pain, establishing individual management plans, prescribing according to standard approaches (where these are available), reviewing patients early and frequently, and referring for specialist opinion in the event of non-response. This study suggests that while treatment may be individualised, many patients are not referred to specialists until relatively late in their clinical course, perhaps leading to a delay in providing optimal treatment.

The activity in pain management is greater among the ‘newly diagnosed’ patients as seen in the pattern of referrals to specialists, where ‘newly diagnosed’ patients were more likely to be referred than ‘established’ patients (81 versus 68%) with a significantly higher referral rate for pain management in the first three years since diagnosis (0.77 versus 0.62 referrals/year). Further development of evidence-based referral guidelines would be helpful to guide the long-term management of this chronic condition and defining what constitutes appropriate levels of referral for all stages of chronic pain management.

Only 8% of all patients in this study were referred to a pain clinic, demonstrating that the majority of pain management is delivered by GPs in primary care. ‘Newly diagnosed’ patients attended their GP surgery for pain-related visits significantly more often than ‘established’ patients, with the most visits occurring in the first year. Further study is needed to determine to what extent the reduction in visit frequency over time is associated with well-controlled pain, requiring only infrequent monitoring, compared with perceived exhaustion of therapeutic options and acceptance by patients of a degree of uncontrolled pain.

The opposite trend was found for non-pain-related visits with ‘established’ patients attending their GP surgery significantly more often than ‘newly diagnosed’ patients. While this may reflect the older age of the ‘established’ group of patients, and consequent co-morbidities, further study is needed to understand the nature of the non-pain-related visits and relationship, if any, with control and manifestation of pain.

As may be expected, GPs appeared to broadly follow the ‘analgesic ladder’ approach to analgesic prescribing. More than half the patients (58%) received NSAIDs. Whilst NSAID use is recommended for OA and LBP (NICE, 2008; 2009), their effectiveness in controlling pain in OA has been shown to be limited to around two to four weeks in most patients (Scott *et al.*, 2000) and there is less evidence of their long-term effectiveness. The lowest effective dose of NSAID should be prescribed for the shortest period of time to control symptoms and the need for long-term treatment should be reviewed periodically (NICE, 2008; 2009; British Medical Association and the Royal Pharmaceutical Society of Great Britain, 2012). Their long-term use in older adults should be limited (British Medical Association and the Royal Pharmaceutical Society of Great Britain, 2012).

Most patients (87%) were prescribed non-opioid analgesics and compound analgesics containing a ‘weak’ opioid (73%). However, in comparison, the number of patients receiving ‘strong’ opioids was considerably lower (17%). There is good evidence for the safety and effectiveness of strong opioids in non-malignant pain (Kalso *et al.*, 2004), and they are agreed to have a role in the early management of back pain (Kalso *et al.*, 2005), but it was difficult to judge how much their use in this study was consistent with the good practice consensus guidance now available from The British Pain Society (2010). Among other important features, these guidelines recommend full and early discussions about potential side effects; close monitoring of dose, effects and possible misuse; their use as part of a wider treatment plan incorporating physical, social and psychological dimensions; the use of modified release preparations where possible; and early referral to specialists in the event of problem drug use. This relative low use of strong opioids may confirm previous findings of GPs’ reluctance to prescribe ‘strong’ opioids because of concerns about effects on patient behaviour, professional competency concerns and degree of belief in opioid effectiveness in chronic pain (McCracken *et al.*, 2008).

The widespread prescription of weak opioids was notable, despite limited evidence of their added benefit over simple analgesics (Li Wan Po and Zhang, 1997). The strategy for reviewing response to medication and adjusting accordingly was not well documented so the opportunity to transfer valuable patient information between healthcare professionals was lost. The results of this study suggest there is a need for additional GP education in the use of analgesics for the long-term management of chronic pain.

There is increasing focus on non-pharmacological approaches to managing chronic pain, and some recent studies have found some of these to be effective in primary care (Heymans *et al.*, 2004; Von Korff *et al.*, 2005; Van Tulder *et al.*, 2006). We found that GPs were reasonably good at referring for these, though might have done so more often. Dissemination and implementation of effective non-pharmacological interventions is an important approach in primary care, in addition to prescribing. The Scottish Intercollegiate Guidelines Network (SIGN) new guidelines on the Management of Chronic Pain address this (SIGN, 2013).

## Conclusion

These results from a retrospective study of a cohort of patients with chronic OA pain or LBP offer a useful picture, previously unavailable, of the management of this resource-intensive group of patients. It seems that prescribing is complex, often involving the management of side effects, and does not conform to a particular pattern. The further development of evidence-based guidelines for primary care treatment and referral for chronic pain could be of great benefit. The need for further research in primary care is therefore apparent, and so too is the need for more education on chronic pain for primary care professionals.
